# Predictor of cognitive impairment: metabolic syndrome or circadian syndrome

**DOI:** 10.1186/s12877-023-03996-x

**Published:** 2023-07-04

**Authors:** Yang Liu, Boying Zang, Jinang Shao, Ning Ning, Lixia He, Yanan Ma

**Affiliations:** 1 Department of Biostatistics and Epidemiology, School of Public Health, China Medical University, No.77 Puhe Road, Shenyang North New Area, Shenyang, 110122 Liaoning Province China; 2 Department of Preventive Medicine, School of Public Heath, North China University of Science and Technology, Tangshan, Hebei China; 3 Division of Molecular and Cellular Oncology, Brigham and Women’s Hospital, Dana-Farber Cancer Institute, Harvard Medical School, Boston, 02215 USA

**Keywords:** Metabolic syndrome, Circadian syndrome, Cognitive impairment, Chinese

## Abstract

**Background:**

It was reported that metabolic syndrome increases the risk for cognitive impairment and circadian rhythm may influence cognition behavior. Identifying the potential risk factors is essential to screen individuals with neuronal dysfunction, neuronal loss, and cognitive decline and prevent cognitive impairment and dementia development.

**Methods:**

We clarified participants by the presence of metabolic syndrome (MetS) and circadian syndrome (CircS) and employed three multivariable Generalized Estimating Equation (GEE) models to control the potential confounding factors and estimate the β values for cognitive function using as referents those had neither MetS nor CircS at baseline. The cognitive function consists of episodic memory and executive function was estimated via the modified Telephone Interview for Cognitive Status (TICS) every two years until 2015.

**Results:**

The mean age of the participants was 58.80 (8.93) years and 49.92% (male). The prevalence of MetS and CircS was 42.98% and 36.43%, respectively. 1,075 (11.00%) and 435 (4.45%) participants had either MetS or CircS alone and 3,124 (31.98%) had both CircS and MetS. Participants with both MetS and CircS compared with normal had a significantly decreased cognitive function score during the 4-years cohort (β = -0.32, 95% CI: -0.63, -0.01) with the complete model, as well as among participants who suffered from CircS alone (β = -0.82, 95% CI: -1.47, -0.16), while not among participants with MetS alone (β = 0.13, 95% CI: -0.27, 0.53). Specifically, compared with the normal population a significantly lower score was discovered in the episodic memory (β = -0.51, 95% CI: -0.95, -0.07), while slightly lower in executive function (β = -0.33, 95% CI: -0.68, -0.01) among individuals with CircS alone.

**Conclusions:**

Individuals with CircS alone or both MetS and CircS have a high risk of cognitive impairment. The association was even stronger in participants with CircS alone than those with both MetS and CircS, suggesting CircS probably have a stronger association with cognitive functioning than MetS and could be a better predictor for cognitive impairment.

**Supplementary Information:**

The online version contains supplementary material available at 10.1186/s12877-023-03996-x.

## Introduction

With improved medical technology and better quality of life, mortality in early life has been dramatically reduced, while it also leads to the rapid aging of populations currently occurring worldwide [1]. Aging accelerates the impairment of cognitive function [1], which declines the quality of life and poses a significant socioeconomic burden. Nowadays, 55.2 million people are suffered from dementia, and this number is calculated to increase 1.5 times by 2050 [2]. To reduce the number of individuals with dementia, identifying its potential risk factors is essential to screen individuals with neuronal dysfunction, and neuronal loss, and prevent cognitive decline and dementia.

The “Metabolic syndrome” (MetS), also known as metabolic syndrome X and cardiometabolic dysfunction, prevalent in developing and developed countries, is a cluster of components that reflect overnutrition, sedentary lifestyles, and excess adiposity [3]. Circadian rhythm is closely related to physiology and behavior and plays an essential role in metabolism [4]. Serving as the basis for an intracellular timekeeping system, the clock genes such as *CLOCK*, Basic Helix-Loop-Helix ARNT Like 1 (*BMAL1*), Period (*Per1* and *Per2*), and Cryptochrome (*Cry1* and *Cry2*) were shown directly related to the MetS in humans and rodents [5]. A loss of function of these clock genes attenuates abnormal circadian rhythm, resulting in the development of the critical components of the MetS, including obesity, hyperglycemia, hyperinsulinemia, hepatic steatosis, and dyslipidemia [6]. The circadian disruption also leads to other comorbidities, such as “Circadian syndrome” (CircS), a new concept proposed by Zimmet et al. [7]. CircS is regarded as one of the important underlying aetiological factors of the MetS and should be recognized together with the MetS cluster and the comorbidities, including sleep disturbances and depression.

Existing evidence indicates MetS is substantially related to a higher cardiovascular disease risk [8] and type 2 diabetes mellius (T2DM) [9, 10]. More complicatedly, cognitive impairment and MetS are not well-established in different age populations and MetS severity. Mid- or late-life MetS has been reported to contribute to developing dementia or cognitive decline, primarily in those with a high level of inflammation [11]. However, MetS and cognitive function might not be positively correlated for the elderly (aged 85 years and older) [12]. In a cross-sectional study with 2,150 subjects aged 60–90 years who scored over 23 points in Mini-Mental State Examination (MMSE), Buyo et al. [13] also drove the conclusion that only attention but not global cognitive function may be impaired by MetS. A cross-sectional study from the Genetics of Brain Structure (GOBS) dataset including 776 Mexican- American adults, as an early adult, young adult, and middle age groups, indicated that the posterior cerebellum emerged as the region most significantly associated with MetS individual components, but advised the future studies should consider the differential effects of age and sex [14]. Reversely, another cross-sectional sample including 112 mid-aged participants (mean age of 50 years) demonstrated MetS was related to cognitive dysfunction, but MetS severity was a better predictor compared with MetS components [15]. It was suggestive that distinguishing and intervening the severity of MetS might help support cognitive functioning. MetS may cause ill effects through a variety of mechanisms on cognitive dysfunction and brain abnormalities, including the vascular stress, neuroinflammation, and dysfunctional lipid metabolism in the brain [16]. There are several factors responsible for the lack of consistency, including the selection of cognitive domains, the quality of tests, the population, the experimental design, and the difficulty of uncoupling the impact of individual factors. Therefore, appropriate adjusted models for the impact of MetS on cognitive function need further exploration. Meanwhile, the connection between circadian and cognition is also unclear. CircS may influence cognition behavior, including alertness, attention, memory, and higher-order executive functions [17]. CircS and cognition impairment are more evident as people age which may be induced by sleep disorders [18]. For example, Merlino et al. [19] found that insomnia was not related to cognitive decline while excessive daytime sleepiness significantly was. The circadian rhythm was also related to Alzheimer’s disease and dementia [20]. However, in another longitudinal study conducted on 5693 individuals who suffered from long-term insomnia and 28,465 participants without, Chen et al. [21] found an elevated dementia risk among the participants with insomnia and prescribed hypnotics. It also indicated that individuals, suffering from long-term insomnia and aged 50–65 years, had an elevated dementia risk than those older than 65 years. The current evidence between circadian and cognition behavior is insufficient and inconsistent, and the connection between MetS/CircS and cognition is unclear and still needs more studies to clarify.

Thus, it is imperative to understand how MetS/CircS impacts cognition and if cognitive impairment could be predictable by MetS/CircS. Given the disparate results of current studies, we aim to estimate whether MetS and CircS statuses were related to cognitive function during a 4-year follow-up in a large-scale cohort study using three multivariable models to control the potential confounding factors. We hypothesize that a standardized model with appropriate confounding factors control would predict cognitive performance beyond the individual component of MetS/CircS.

## Methods

### Study population

CHARLS is a nationwide prospective cohort representing the middle-aged and elderly population in China. Participants aged ≥ 45 were enrolled. Candidates’ samples were established via geographic information system (GIS) software and multistage probability sampling method, covering 450 villages and communities in 150 counties and districts nationwide.

**Fig. 1:**
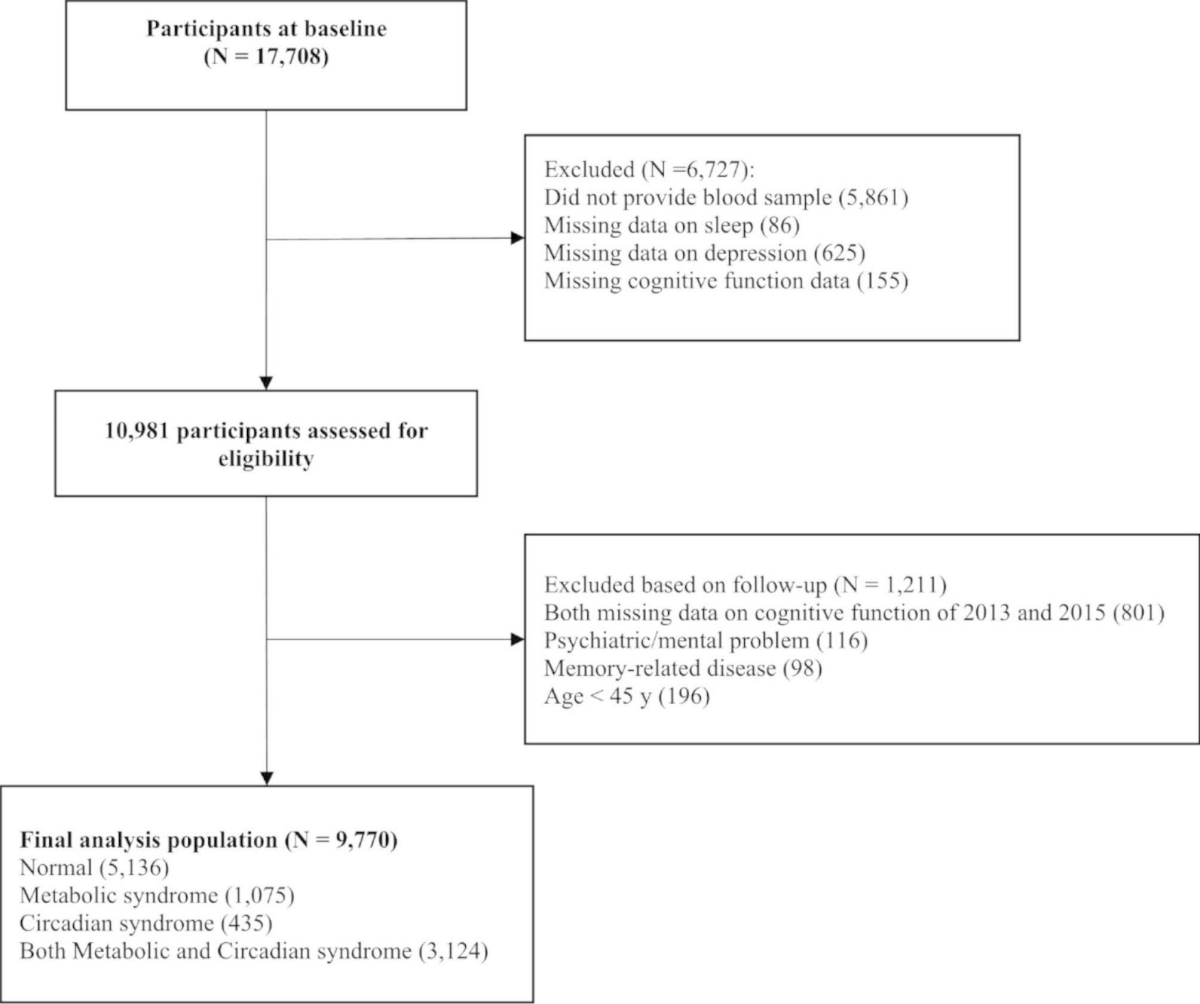
Flow diagram for participants included in the study

Overall, 17,708 participants who achieved face-to-face computer-assisted personal interviews (CAPI) in 10,257 households had an overall response rate of 80.5% at baseline. Meanwhile, follow-up surveys were conducted every 2 years [22, 23]. The current study first included 10,981 participants who provided blood samples and contained complete information on sleep, depression, and cognitive function. And further excluded 1,211 participants who lost data on cognitive function during follow-ups (n = 801), were physician-diagnosed with a mental problem, brain damage, or memory-related disease (n = 214), age < 45 years (n = 196). In the end, 9,770 adults were enrolled in the final analyses (Fig. 1). This study abided by the guidelines of the Declaration of Helsinki and the protocol was approved by the Ethical Review Committee of Peking University (approval number: IRB 00001052–11,015) and each participant provided written informed consent.

### Metabolic syndrome and circadian syndrome

Medically trained personnel from the Chinese Center for Disease Control and Prevention collected and stored venous blood samples at -80 °C. Capital Medical University’s Youanmen Center for Clinical Laboratory measured various biomarkers, including cholesterol (total, HDL, and LDL) and glucose [24]. During the testing of study samples, quality control samples were used daily (from February 2013 to June 2013). All results derived from these quality control samples were within the target range (mean ± 2 standard deviation (SD)).

Blood pressure was measured by medical staff with an electronic blood pressure monitor (Omron™ HEM-7112). The mean of three measurements, made 45 s apart, was calculated. Systolic blood pressure above 140 mmHg, diastolic blood pressure above 90 mmHg, or usage of antihypertensive medicine was defined as hypertension [25].

The Center for Epidemiologic Studies-Depression scale (CES-D) was used to estimate depression symptoms. CES-D score above 9 was considered a sign of depression [26].

MetS was based on harmonizing the metabolic syndrome. The occurrence of any 3 components composed a diagnosis of metabolic syndrome [27]. Dyslipidemia medicine usage was collected according to western or Chinese medications used by self-report.

The circadian syndrome was defined according to the presence of any 4 components: 5 components mentioned above; short sleep (< 6 h per day); depression symptoms [28] (Table 1**)**.

**Table 1:** Definition of metabolic syndrome and circadian syndrome

Measure	Categorical cut point	Included in MetS	Included in CircS
Elevated waist circumference	Population- and country- specific definitions (≥ 85 cm in men, ≥ 80 cm in women)	√	√
Elevated triglycerides (drug treatment for elevated triglycerides is an alternate indicator)	≥ 150 mg dL^− 1^ (1.7 mmol L^− 1^)	√	√
Reduced HDL-C (drug treatment for reduced HDL-C is an alternate indicator)	< 40 mg dL^− 1^ (1.0 mmol L^− 1^) in men; < 50 mg dL^− 1^ (1.3 mmol L^− 1^) in women;	√	√
Elevated blood pressure (antihypertensive drug treatment in a patient with a history of hypertension is an alternate indicator)	Systolic ≥ 130 and/or diastolic ≥ 85 mmHg	√	√
Elevated fasting glucose (drug treatment of elevated glucose is an alternate indicator)	≥ 100 mg dL^− 1^	√	√
Short sleep	< 6 h day^− 1^		√
Depression symptom	10 item CES-D score ≥ 10		√
Definition criteria		≥ 3 components	≥ 4 components

### Cognitive function Assessment

Using the modified Telephone Interview for Cognitive Status (TICS), cognitive function was assessed at baseline and follow-up. Briefly, the cognitive function was investigated three times (2011, 2013, and 2015, respectively) and comprised two dimensions: executive function and episodic memory [29]. Executive function was accessed by numerical ability (subtracting 7 from 100 five times in a row), time orientation (e.g., weeks, months, days, seasons, and years), and visuo-construction (figure drawing). Scores for these domains were combined into one score from 0 to 11 points [23]. To evaluate episodic memory, words were recalled immediately (within 2 min) and delayed (4–10 min). Afterward, interviewers read 10 random words to participants and asked them to repeat as many as they could. The episodic memory scores depended on the number of words correctly repeated, ranging from 0 to 20. This combined score of 0 to 31 represents the overall cognitive function [30].

### Covariates

In the current study, the following covariates were selected at the baseline survey: anthropometer (BMI); demographic factor (age, gender, residence, marital status); socioeconomic status (household income per year and educational levels,); lifestyle factor (physical activity, smoking status, and alcohol consumption); and history of chronic disease (hypertension, T2DM, stroke, heart disease, chronic kidney disease, and cancer).

BMI (kg/m^2^) was measured based on a uniform formula: weight (kg) / height^2^ (m^2^). Participants with self-reported T2DM, receiving T2DM treatment, meeting the American Diabetes Association (ADA) T2DM criteria (FPG ≥ 126 mg dL^− 1^ [7.0 mmol L^− 1^]) or participants with physician-diagnosed T2DM were defined as suffering from T2DM [31].

### Statistical analysis

We described and compared the distribution of baseline characteristics according to MetS and CircS statuses utilizing mean ± standard deviation and one-way ANOVA for continuous variables, while number (proportions) and Chi-square tests were used for categorical variables. Multivariable Generalized Estimating Equation (GEE) models were utilized to estimate whether MetS and CircS statuses were associated with cognitive function in the 4-years cohort. Three multivariable models were constructed to control potential confounding factors. Model 1 was adjusted for follow-up time, age, age^2^, gender, marital status, residence, household income per year, and education level. Model 2 was further adjusted for BMI, smoking status, drinking status, and physical activity based on Model 1. Model 3 (fully adjusted model) incorporated the types of major chronic diseases in Model 2.

Interaction analyses were performed for MetS and CircS statuses to access whether anthropometric factors, demographic factors, socioeconomic status, lifestyle, and chronic disease modified the association between MetS and CircS statuses and cognitive function.

### Sensitivity analyses

To further verify the stability of the results, the following sensitivity analyses were performed: (1) Stratified analyses were conducted in age (45–60, > 60 years), BMI (< 24, ≥ 24 kg/m^2^), gender (male, female), residence (rural, urban), education years (< 9, ≥ 9 years), household income per year (≤ 30,000, > 30,000 yuan), marital status (live with a spouse, live without a spouse), smoking status (non-smoker, ever and current smoker), alcohol consumption (never, abstainer and current drinker), physical activity (non and mild, moderate and vigorous) and history of major chronic diseases (no, yes). (2) Participants were divided into normal and disease groups (suffering from MetS or CircS), and the fully adjusted model was conducted to assess the association between the binary of participants and cognitive function. Simultaneously, interaction and stratified analyses were conducted. (3) After we excluded those with an extremely low overall cognitive function score (< mean-2SD) at baseline, the association between MetS and CircS statuses and cognitive function was checked with the fully adjusted model (N = 9602). (4) Association between MetS and CircS statuses and cognitive function was further confirmed by a fully adjusted model after excluding those diagnosed with brain damage, mental retardation, or memory-related diseases in 2013 or 2015 (N = 9596). (5) In addition, the missing data were imputed using multiple imputations, using 5 replications and chained equations in R Multiple imputations (MI). We further excluded the participants who tended to have the habit of smoking and re-analyzed the results.

Statistical analyses were performed using the R statistical package (http://www.R-project.org; version 3.6.3) and Empower (R) software (www.empowerstats.com, X&Y Solutions, Inc., Boston MA, USA). There was statistical significance at a two-sided *P* value below 0.05.

## Results

### Characteristics of participants

The characteristics of participants enrolled in the current study after inclusion and exclusion were reported in Table 2. The participants were 58.80 (SD 8.93) years and 49.92% (male) in 2011. The prevalence of MetS and CircS was 42.98% and 36.43%, respectively. In sum, 1,075 (11.00%) and 435 (4.45%) participants had either MetS or CircS alone and 3,124 (31.98%) had both CircS and MetS. The participants who suffered from MetS and/or CircS tended to be older, allocated in inferior socioeconomic status, had higher BMI, dropped out from drinking and smoking, lacked sleep, fell into depression, and were hunted by a more heavily chronic disease burden. In addition, participants who suffered from CircS alone were accompanied by the lowest cognitive function at baseline.

**Table 2:** Baseline characteristics of participants overall and by MetS and/or CircS status (n = 9,770)

Variable	Age	MetS and/or CircS Status				
		**Normal** **(n = 5,136)**	**MetS alone** **(n = 1,075)**	**CircS alone** **(n = 435)**	**Both MetS and CircS** **(n = 3124)**	*P*-value
**Age (mean ± SD)**	58.80 ± 8.93	58.39 ± 9.04	58.18 ± 8.81	60.22 ± 8.91	59.48 ± 8.74	< 0.001
**BMI (kg/m** ^**2**^ **)**		22.11 ± 3.35	24.99 ± 3.42	22.63 ± 3.68	25.67 ± 3.79	< 0.001
**Male (n, %)**	59.63 ± 8.94	2776 (54.07)	551 (51.26)	178 (40.92)	1078 (34.51)	< 0.001
**Rural inhabitant (n, %)**	58.79 ± 8.93	2776 (54.07)	551 (51.26)	178 (40.92)	1078 (34.51)	< 0.001
**Live with spouse (n, %)**	58.16 ± 8.52	4402 (85.71)	956 (88.93)	327 (75.17)	2612 (83.61)	< 0.001
**Household income > 30,000 per year (n, %)**	56.54 ± 8.18	1283 (26.75)	298 (30.10)	66 (16.58)	752 (26.32)	< 0.001
**Education (n, %)**						< 0.001
Illiterate	61.21 ± 8.92	2282 (44.44)	406 (37.80)	266 (61.15)	1516 (48.54)	
Primary school	59.22 ± 8.60	1202 (23.41)	228 (21.23)	84 (19.31)	693 (22.19)	
Middle school	54.87 ± 7.80	1088 (21.19)	274 (25.51)	61 (14.02)	605 (19.37)	
High school or above	55.24 ± 7.91	563 (10.96)	166 (15.46)	24 (5.52)	309 (9.89)	
**Smoking status (n, %)**						< 0.001
Non-smokers	58.26 ± 8.99	2839 (55.29)	640 (59.53)	270 (62.07)	2185 (69.94)	
Ex-smokers	61.75 ± 9.24	434 (8.45)	107 (9.95)	27 (6.21)	282 (9.03)	
Current smokers	59.01 ± 8.56	1862 (36.26)	328 (30.51)	138 (31.72)	657 (21.03)	
**Drinking status (n, %)**						< 0.001
Never drank	58.63 ± 9.07	2808 (54.68)	607 (56.52)	265 (60.92)	2077 (66.49)	
Abstainers	61.84 ± 8.61	404 (7.87)	76 (7.08)	41 (9.43)	270 (8.64)	
Current drinkers	58.34 ± 8.62	1923 (37.45)	391 (36.41)	129 (29.66)	777 (24.87)	
**Physical activity (n, %)**						< 0.001
None	61.17 ± 9.74	174 (8.08)	53 (10.60)	21 (10.77)	168 (12.34)	
Mild	60.69 ± 9.56	414 (19.23)	114 (22.80)	47 (24.10)	388 (28.51)	
Moderate	58.31 ± 8.73	623 (28.94)	169 (33.80)	66 (33.85)	433 (31.81)	
Vigorous	57.01 ± 8.03	942 (43.75)	164 (32.80)	61 (31.28)	372 (27.33)	
Missing	58.89 ± 8.93	2983	575	240	1763	
**Hypertension (n, %)**	60.39 ± 9.15	1687 (32.99)	607 (56.62)	218 (50.35)	2109 (67.68)	< 0.001
**Cancer**	58.40 ± 9.64	37 (0.72)	13 (1.21)	10 (2.31)	30 (0.97)	0.006
**Heart diseases**	60.95 ± 8.80	460 (9.01)	106 (9.91)	80 (18.43)	549 (17.69)	< 0.001
**Stroke**	62.95 ± 8.75	68 (1.33)	13 (1.21)	12 (2.76)	93 (2.99)	< 0.001
**Type 2 Diabetes Mellitus**	59.81 ± 8.94	524 (10.28)	219 (20.56)	68 (15.81)	938 (30.29)	< 0.001
**Chronic kidney disease**	58.48 ± 8.43	345 (6.76)	47 (4.40)	45 (10.42)	200 (6.43)	< 0.001
**Elevated waist circumference**	58.51 ± 8.73	1550 (30.18)	786 (73.12)	202 (46.44)	2585 (82.75)	< 0.001
**Elevated serum triglycerides**	58.14 ± 8.58	252 (4.91)	325 (30.23)	38 (8.74)	2001 (64.05)	< 0.001
**Reduced serum HDL-C**	58.24 ± 8.67	683 (13.30)	560 (52.09)	119 (27.36)	2450 (78.43)	< 0.001
**Elevated blood pressure**	60.18 ± 9.17	2034 (39.60)	771 (71.72)	262 (60.23)	2502 (80.09)	< 0.001
**Elevated plasma glucose**	59.32 ± 8.85	1898 (36.95)	783 (72.84)	249 (57.24)	2675 (85.63)	< 0.001
**Short sleep**	60.44 ± 9.00	1239 (24.12)	0 (0.00)	435 (100.00)	1202 (38.48)	< 0.001
**Depression**	59.62 ± 8.92	1677 (32.65)	0 (0.00)	435 (100.00)	1496 (47.89)	< 0.001
**Baseline cognitive function (mean ± SD)**						
Episode memory	58.80 ± 8.93	5.71 ± 2.67	6.19 ± 2.57	4.61 ± 2.71	5.48 ± 2.72	< 0.001
Executive function	58.80 ± 8.93	6.60 ± 3.78	7.25 ± 3.80	5.46 ± 3.49	6.43 ± 3.71	< 0.001
Overall cognitive function	58.80 ± 8.93	12.31 ± 5.35	13.44 ± 5.33	10.07 ± 5.04	11.90 ± 5.37	< 0.001

### Association between baseline metabolic syndrome and circadian syndrome status and cognitive function at follow-up

In the fully adjusted model adjusted for follow-up time, anthropometric (BMI); demographic factors, socioeconomic status, lifestyle, and health conditions, participants with both MetS and CircS compared with normal had a significantly decreased cognitive function score during 4-year cohort (β = -0.32, 95% CI: -0.63, -0.01), as well as among participants who suffered from CircS alone (β = -0.82, 95% CI: -1.47, -0.16), while not among participants with MetS alone (β = 0.13, 95% CI: -0.27, 0.53) (Table 3). Meanwhile, compared with the normal population, a significantly lower score was noted in the episodic memory dimension (β = -0.51, 95% CI: -0.95, -0.07), while slightly lower in the executive function dimension (β = -0.33, 95% CI: -0.68, -0.01) among participants with CircS alone. Although no significant association was revealed in episodic memory dimension (β = -0.22, 95% CI: -0.44, 0.01) and executive function dimension (β = -0.14, 95% CI: -0.30, 0.03) in the population who suffered from both MetS and CicS, it is worth heeding to the evident risks in a longer follow-up period compared with normal participants. A positive association was not demonstrated among participants with MetS alone neither in the episodic memory dimension (β = 0.05, 95% CI: -0.23, 0.32) nor executive function dimension (β = 0.12, 95% CI: -0.09, 0.33) (Table 4).

**Table 3:** Association between MetS and/or CircS status and cognitive function (n = 9,770)

Overall cognitive function	MetS and/or CircS status			
	Normal (n = 5,136)	MetS/CircS (n = 4,634)		
		MetS alone (n = 1,075)	CircS alone (n = 435)	Both MetS and CircS (n = 3124)
Model 1	Ref	0.49 (0.24, 0.73) *	-0.91 (-1.27, -0.54) *	-0.13 (-0.30, 0.04)
Model 2	Ref	0.07 (-0.31, 0.46)	-0.83 (-1.48, -0.18) *	-0.35 (-0.65, -0.06) *
Model 3	Ref	0.13 (-0.27, 0.53)	-0.82 (-1.47, -0.16) *	-0.32 (-0.63, -0.01) *

Model 1: Adjusted for follow-up time, age, age^2^, gender, residence, educational level, household annual income, and marital status;

Model 2: Adjusted for covariates in Model 1 + body mass index, smoking status, drinking status, and physical activity;

Model 3: Adjusted for covariates in Model 2 + hypertension, T2DM, stroke, heart disease, chronic kidney disease, and cancer

*P < 0.05

**Table 4:** Association between MetS and/or CircS status and executive function and episodic memory (n = 9,770)

Cognitive domains	MetS and/or CircS status			
	Normal (n = 5,136)	MetS/CircS (n = 4,634)		
		MetS alone (n = 1,075)	CircS alone (n = 435)	Both MetS and CircS (n = 3124)
**Executive function**				
Model 1	Ref	0.27 (0.14, 0.39) *	-0.43 (-0.64, -0.23) *	-0.04 (-0.13, 0.05)
Model 2	Ref	0.08 (-0.13, 0.28)	-0.36 (-0.71, -0.02) *	-0.18 (-0.34, -0.02) *
Model 3	Ref	0.12 (-0.09, 0.33)	-0.33 (-0.68, 0.01)	-0.14 (-0.30, 0.03)
**Episodic memory**				
Model 1	Ref	0.25 (0.08, 0.43) *	-0.45 (-0.69, -0.20) *	-0.11 (-0.23, 0.01)
Model 2	Ref	0.02 (-0.24, 0.29)	-0.50 (-0.93, -0.06) *	-0.23 (-0.44, -0.02) *
Model 3	Ref	0.05 (-0.23, 0.32)	-0.51 (-0.95, -0.07) *	-0.22 (-0.44, 0.01)

Model 1: Adjusted for follow-up time, age, age^2^, gender, residence, educational level, household income per year, and marital status;

Model 2: Adjusted for covariates in Model 1 + body mass index, smoking status, drinking status, and physical activity;

Model 3: Adjusted for covariates in Model 2 + hypertension, T2DM, stroke, heart disease, chronic kidney disease, and cancer

*P < 0.05

**Fig. 2:**
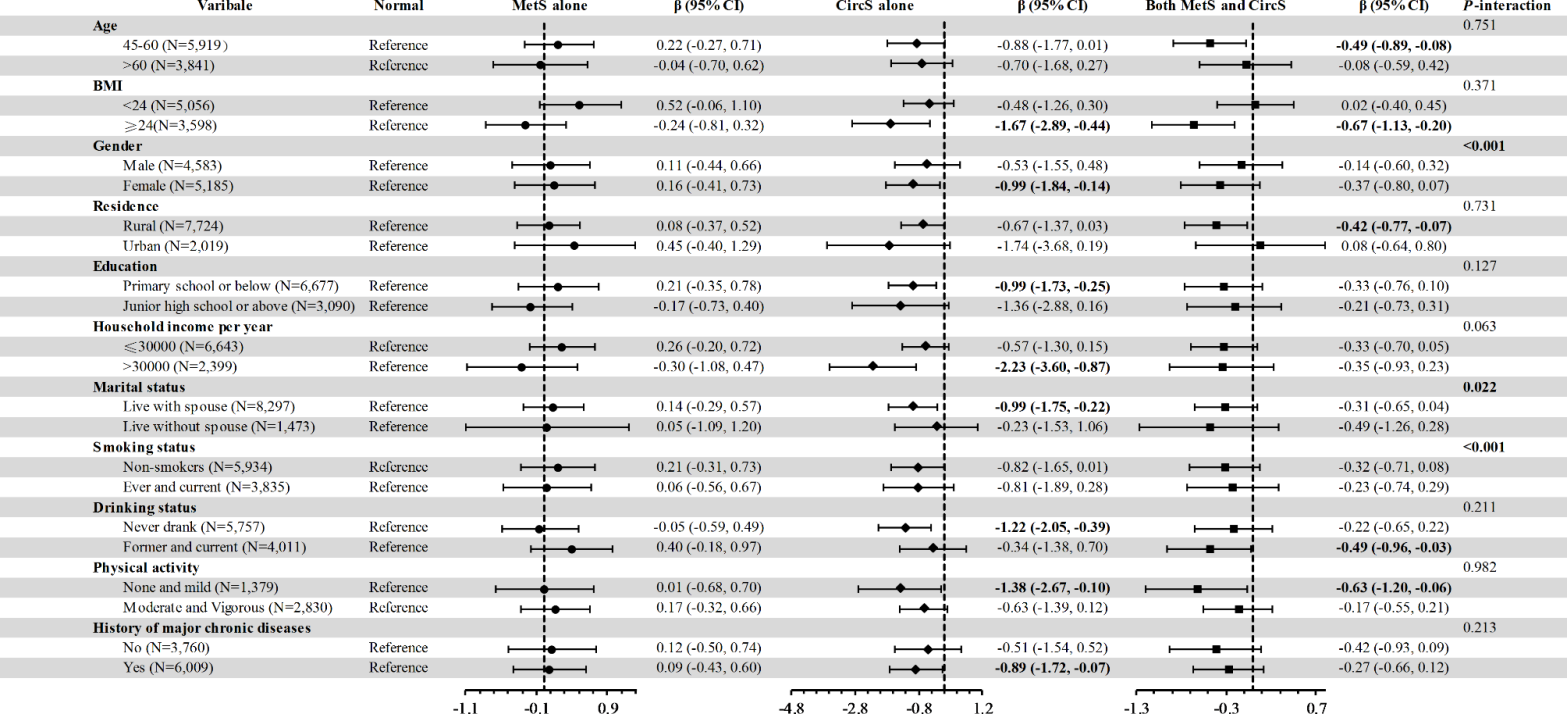
Difference in cognitive function for MetS and/or CricS status compared with the normal in fully adjusted models

The results of interaction analyses shown in Fig. 2 found that gender, marital status, and smoking status were distinguished acting as effect modifiers (*P*-interaction < 0.05). According to the repeated measurement results, it’s worth mentioning that the cognitive decline in females was more remarkable than in males. However, for smoking status and marital status, the tendencies of change in cognitive function were not dramatically twisted by different statuses (Fig. 2).

### Sensitivity analyses

When conducting interaction and stratified analyses, there were no significant interactions in most of the variables between MetS and/or CricS status and cognitive function, except gender, marital status, and smoking status. However, the significant interactions in gender, marital status, and smoking status were likely to contribute to the MetS alone. The participants with CircS alone or both MetS and CircS showed the same trends, which were consistent with our core results of significant correlation between CircS alone or both MetS and CircS and cognitive function, but not between MetS alone and cognitive function (Fig. 2). When we combined participants having MetS or/and CircS and compared them with normal, participants with MetS or/and CircS displayed lower cognitive function after adjusting for all confounding factors (Supplementary Table 1). Although the differences were not significant, the tendencies of all confounders in the fully adjusted model kept in the line with the negative influence of MetS or/and CircS in cognitive function. Stratification and interaction analysis further verified the reliability of these results with the emerging risks in each subgroup as well as significant differences derived from higher BMI, rural residents, and lack of physical activity population. And gender, smoking, and drinking also were discovered as effect modifiers of MetS or/and CircS compared with the normal population but the declining trend did not change (Supplementary Table 2). Furthermore, we performed the fully adjusted models after excluding those with an extremely low overall cognitive function score (< mean-2SD) at baseline. Although some of the results were without statistical significance, a general trend kept declining among CircS alone and both MetS and CircS participants. Similarly, we did not see an association between MetS alone and cognitive function after excluding those with an inefficient cognitive function (Supplementary Table 3). We further excluded those diagnosed with brain-related diseases during follow-up periods. The association between MetS alone and cognitive function was negative, while the association between CircS alone, combined MetS and CircS, and cognitive function was preserved significantly (Supplementary Table 4). The results were still kept in line with primary results after MI (Supplementary Table 5). The results were not materially changed after excluding the participants who had the habit of smoking (Supplementary Table 6).

## Discussion

In this longitudinal study of middle-aged and elderly Chinese, data was gathered from a nationally representative sample, our results with a fully adjusted model showed that the combination of MetS and CircS was significantly associated with cognitive impairment. Meanwhile, individuals with CircS alone also had a high risk of cognitive decline. The association was even stronger in participants with CircS alone than those with both MetS and CircS, suggesting CircS components probably have a stronger association with cognitive functioning than MetS components. Besides, the female exhibited more significant declines in cognitive function than the male. However, our fully adjusted model did not reveal a significant association between MetS alone and cognitive decline.

According to our knowledge, this is the first research study to investigate the connection between the CircS cluster and cognitive impairment. The available proof showed that sleep deprivation was associated with cognitive function, manifesting in memory, attention, and processing speed of shift workers [32]. Similar to our results, Yaffe et al. [20] concluded that circadian disturbance increased the risk of cognitive decline through both sleep-dependence and sleep-independence processes. Moreover, a large-scale study with 91,105 participants showed that the disruption of circadian rhythms was related to higher neuroticism scores (incident rate ratio 1.01, 95% CI 1.01–1.02) [33]. We extended the previous results by adjusting for covariates with three different multivariable models and replacing variable sleep deprivation with variable CircS, which was assessed in a large-scale sample. CircS is a larger concept that is not only connected with circadian rhythms but also related to the components of MetS. Our results showed evidence that CircS is strongly related to cognitive impairment with three adjusted models. We speculate that CircS may be one of the most important influencing factors. People with metabolic syndrome, representing metabolic dysfunctional status, are more likely to have abdominal obesity rather than general obesity measured by BMI and have IR (insulin resistance), both are associated with metabolic dysregulation [27]. Meanwhile, abdominal obesity shows a robust link with IR which is one of the most important causes of diabetes a severe metabolic disease [34]. Thus, a study considered it necessary for both diagnostic criteria of MetS and primary care to measure waist circumstances to effectively screen the potential metabolism-disordering population although a more precise cut-off point will have to be made [27].

As a result of limited research, the underlying mechanism of CircS and cognitive function remains unclear. Different mechanisms of cognitive decline led by circadian rhythms have been proposed. For instant, it was indicated that the activity of cortical and subcortical brain regions associated with cognition, including the thalamus, anterior hypothalamus, and locus coeruleus of the brainstem, could be affected by circadian rhythms [35]. Furthermore, the interaction of clock dysfunction and neuropsychiatric disease was also suggested [5], due to a gene encoding the molecular clock core component having multiple single nucleotide polymorphisms (SNPs). The involved mechanism merits further investigation, and more longitudinal studies are necessary to provide more insight into the association between CircS and/or combined with MetS and cognitive function.

Intriguingly, with the fully adjusted model, we could not see the significant impairment of cognitive function associated with MetS, which has been reported in previous studies. In a 5-year prospective observational study with 2,632 participants’ mean age of 74 years, it showed that elder participants with the MetS have a higher risk of cognitive impairment than those without the MetS [11]. In addition, excluding those participants with overt diabetes, frank hypertension, or clinically significant hyperlipidemia in this study, the multivariable-adjusted models with different covariates likely attributed to the different results. Although this study included some covariates associated with cognitive impairment in the multivariate-adjusted logistic regression model, such as depression score and baseline cognitive score, the other covariates also play essential roles in cognitive impairment. According to our study, MetS was significantly associated with cognitive function found in Model 1 but was not seen in other adjusted models, indicating the critical influence of the added covariates on cognitive function in Model 2 and Model 3. The body mass index (BMI), hypertension, heart disease, and diabetes were associated with cognitive function in previous studies [36–38]. Besides, hyperglycemia was the primary predictor of cognitive decline, which was also related to diabetes [39]. Avadhani et al. [37] found that higher HbA1c was related to lower cognitive performance scores in MetS patients. Therefore, it is vital to include all covariates associated with cognitive impairment to avoid any biases.

In addition, we performed a further investigation in two major cognitive domains, executive function, and episodic memory, with the three multivariable-adjusted models. We found that the positive association in executive function was not held between MetS alone, CircS alone, or both Mets and CircS with the fully adjusted model. However, a strong association with CircS alone was held among different adjusted models for episodic memory, consistent with the significant association between CircS alone and cognitive function. The stability of these results was also further verified by the sensitivity analyses.

These findings support the hypothesis that cognitive performance would be better predictable by CircS or the combination of MetS and CircS beyond individual components of MetS/CircS, which could be of great value in the prevention of cognitive impairment and will be of great significance to public health. Our study on the association between MetS/CircS and cognition was based on a large-scale representative sample and adjusted for all potential confounders, which enhanced the robustness of our analyses and the unbiases of the results. However, several limitations of our study may affect the interpretation of our results. Firstly, our study was only based on the China Health and Retirement Longitudinal Study (CHARLS), so the results may not be able to be interpreted for the worldwide population. Secondly, an assessment of cognitive function at baseline and during follow-up by using the modified Telephone Interview for Cognitive Status (TICS) approach, which might produce different results from an in-person interview. Thirdly, only 4-year follow-up was performed in this study. Some trends in changes without statistical significance might be observed with a longer follow-up. Fourthly, the current study would be better if we could include the gray matter volume indicator. Unfortunately, the database in this study did not collect gray matter volume data. Fifthly, the broad definition of covariates such as diabetes and hypertriton was applied in the current study according to previous studies which focused on the association between chronic diseases and cognition [25, 40], which may result in potential inaccuracy although it was widely used. Sixthly, the CHARLS we used is a nationwide prospective cohort representing the middle-aged and elderly population in China, which may lead to a limitation in the evidence applicable population. Therefore, further investigation and more longitudinal research are needed to verify the association between CircS and/or combined with MetS and cognitive function.

## Conclusion

In conclusion, our study conducted in this nationally representative longitudinal survey provides novel evidence of the association between cognitive function and the synchronous or respective presence of the MetS and CircS. CircS has a stronger association with cognitive function than MetS, suggesting that CircS may be a great predictor of cognitive impairment. Our study supports the results of previous studies that the circadian rhythms altered cognitive function and extends it to the predictive efficacy of CircS on cognitive impairment. Consequently, any preventative or treatment approaches that could decrease the risk of cognitive impairment would have a tremendous impact on the quality of life of the elders.

## Electronic supplementary material

Below is the link to the electronic supplementary material.

Supplementary Material 1

## Data Availability

The data that support the findings of this study are available from the open CHARLS database.

## Abbreviations

MetS: Metabolic syndrome
CircS: Circadian syndrome
MMSE: Mini-Mental State Examination
HDL: High-density lipoprotein
HDL-C: High-density lipoprotein cholesterol
LDL: Low density lipoprotein
HR: Hazard ratio
OR: Odds ratio
CI: confidence interval
SD: Standard deviation
RRs: Relative risks
GEE: Generalized Estimating Equation
CES-D: Center for Epidemiologic Studies-Depression scale
CHARLS: The China Health and Retirement Longitudinal Study
GIS: Geographic information system
CAPI: Computer-assisted personal interview
T2DM: Type 2 diabetes mellitus
IR: insulin resistance
