# Supplementary material for: Predictor of cognitive impairment: metabolic syndrome or circadian syndrome
Source: BMC Geriatr. 2023 Jul 4;23:408. doi: 10.1186/s12877-023-03996-x (PMC10318700; doi:10.1186/s12877-023-03996-x)
Supplement: Supplementary file 1 — Supplementary Material 1 [file 12877_2023_3996_MOESM1_ESM.pdf]

Supplementary Table 1 Association between MetS and/or CircS status and executive function and episodic memory (N = 9,770)

Supplementary Table 2 Difference in cognitive function for MetS and/or CricS compared with the normal in fully adjusted models.

Supplementary Table 3 Association between MetS and/or CircS status and cognitive function (overall and domains) in Chinese middle-aged and elderly participants based on the generalized estimating equation after excluding those with an extremely low overall cognitive function score (< mean-2sd) at baseline (N=9,602).

Supplementary Table 4 Association between MetS and/or CircS status and cognitive function (overall and domains) in Chinese middle-aged and elderly participants based on the generalized estimating equation after excluding those diagnosed with brain damage, mental retardation, or memory-related diseases in 2013 or 2015 (N=9,596).

Supplementary Table 5 Association between MetS and/or CircS status and cognitive function (overall and domains) in Chinese middle-aged and elderly participants based on the generalized estimating equation after multiple imputation of missing data (N=9,770).

Supplementary Table 6 Association between MetS and/or CircS status and cognitive function (overall and domains) in Chinese middle-aged and elderly participants based on the generalized estimating equation after excluding the participants who had smoking history at baseline (N=5,934).

Supplementary Table 1 Association between MetS and/or CircS status and executive function and episodic memory (N = 9,770)

Cognitive function	MetS and/or CircS status	
	**Normal (n = 5,136)**	**MetS or/and CircS (n = 4,634)**
**Executive function**		
Model 1	Ref	-0.00 (-0.08, 0.08)
Model 2	Ref	-0.13 (-0.27, 0.01)
Model 3	Ref	-0.09 (-0.24, 0.06)
**Episodic memory**		
Model 1	Ref	-0.05 (-0.16, 0.05)
Model 2	Ref	**-0.20 (-0.38, -0.01) ***
Model 3	Ref	-0.18 (-0.38, 0.01)
**Overall cognitive function**		
Model 1	Ref	-0.05 (-0.20, 0.10)
Model 2	Ref	**-0.30 (-0.56, -0.03) ***
Model 3	Ref	-0.26 (-0.53, 0.02)

Model 1: Adjusted for follow-up time, age, age^2, gender, residence, educational level, household annual income, and marital status;

Model 2: Adjusted for covariates in Model 1 + body mass index, smoking status, drinking status, and physical activity;

Model 3: Adjusted for covariates in Model 2+ hypertension, T2DM, stroke, heart disease, chronic kidney disease, and cancer.

**P*<0.05

Supplementary Table 2 Difference in cognitive function for MetS and/or CricS compared with the normal in fully adjusted models.

Variable	MetS and/or CircS status		
	**Normal (n = 5,136)**	**MetS and/or CircS (n = 4,634)**	***P* for interaction**
**Age**			0.671
45-60 (N=5,919)	1.0 (Reference)	-0.32 (-0.68, 0.03)	
>60 (N=3,841)	1.0 (Reference)	-0.17 (-0.62, 0.28)	
**BMI**			0.597
<24 (N=5,056)	1.0 (Reference)	0.07 (-0.29, 0.43)	
⩾24(N=3,598)	1.0 (Reference)	**-0.60 (-1.03, -0.17)**	
**Gender**			**<0.001**
Male (N=4,583)	1.0 (Reference)	-0.11 (-0.50, 0.29)	
Female (N=5,185)	1.0 (Reference)	-0.32 (-0.71, 0.07)	
**Residence**			0.568
Rural (N=7,724)	1.0 (Reference)	**-0.32 (-0.63, -0.01)**	
Urban (N=2,019)	1.0 (Reference)	0.09 (-0.57, 0.74)	
**Education**			0.264
Primary school or below (N=6,677)	1.0 (Reference)	-0.30 (-0.68, 0.07)	
Junior high school or above (N=3,090)	1.0 (Reference)	-0.27 (-0.72, 0.18)	
**Household income per year**			0.191
≤30000 (N=6,643)	1.0 (Reference)	-0.20 (-0.53, 0.13)	
>30000 (N=2,399)	1.0 (Reference)	-0.49 (-1.02, 0.04)	
**Marital status**			0.055
Live with spouse (N=8,297)	1.0 (Reference)	-0.26 (-0.56, 0.05)	
Live without spouse (N=1,473)	1.0 (Reference)	-0.31 (-1.00, 0.38)	
**Smoking status**			**0.001**
Non-smokers (N=5,934)	1.0 (Reference)	-0.25 (-0.61, 0.11)	
Ever and current (N=3,835)	1.0 (Reference)	-0.20 (-0.65, 0.24)	
**Drinking status**			**0.026**
Never drank (N=5,757)	1.0 (Reference)	-0.30 (-0.69, 0.08)	
Former and current (N=4,011)	1.0 (Reference)	-0.20 (-0.60, 0.21)	
**Physical activity**			0.735
None and mild (N=1,379)	1.0 (Reference)	**-0.52 (-1.03, -0.02)**	
Moderate and Vigorous (N=2,830)	1.0 (Reference)	-0.14 (-0.47, 0.19)	
**History of major chronic diseases**			0.969
No (N=3,760)	1.0 (Reference)	-0.28 (-0.69, 0.14)	
Yes (N=6,009)	1.0 (Reference)	-0.26 (-0.62, 0.10)	

Supplementary Table 3 Association between MetS and/or CircS status and cognitive function (overall and domains) in Chinese middle-aged and elderly participants based on the generalized estimating equation after excluding those with an extremely low overall cognitive function score (< mean-2sd) at baseline (N=9,602).

**Cognitive function**	**MetS and/or CircS status**			
	**Normal (n = 5,053)**	**MetS/CircS (n = 4,634)**		
		MetS alone (n = 1,060)	CircS alone (n = 423)	Both MetS and CircS (n = 3,066)
**Executive function**				
Model 1	Ref	0.27 (0.14, 0.39)	**-0.45 (-0.66, -0.25) ***	-0.03 (-0.12, 0.05)
Model 2	Ref	0.06 (-0.14, 0.26)	**-0.36 (-0.70, -0.01) ***	**-0.18 (-0.34, -0.02) ***
Model 3	Ref	0.11 (-0.10, 0.32)	-0.32 (-0.67, 0.03)	-0.13 (-0.30, 0.04)
**Episodic memory**				
Model 1	Ref	0.26 (0.09, 0.43)	**-0.47 (-0.72, -0.23) ***	-0.11 (-0.23, 0.01)
Model 2	Ref	0.00 (-0.26, 0.27)	**-0.49 (-0.93, -0.05) ***	**-0.23 (-0.44, -0.02) ***
Model 3	Ref	0.04 (-0.23, 0.31)	**-0.49 (-0.94, -0.05) ***	-0.20 (-0.43, 0.02)
**Overall cognitive function**				
Model 1	Ref	0.49 (0.25, 0.73)	**-0.95 (-1.32, -0.58) ***	-0.12 (-0.29, 0.04)
Model 2	Ref	0.05 (-0.34, 0.44)	**-0.83 (-1.48, -0.17) ***	**-0.35 (-0.64, -0.05) ***
Model 3	Ref	0.12 (-0.28, 0.52)	**-0.80 (-1.46, -0.14) ***	-0.29 (-0.61, 0.02)

Model 1: Adjusted for follow-up time, age, age^2, gender, residence, educational level, household income per year, and marital status;

Model 2: Adjusted for covariates in Model 1 + body mass index, smoking status, drinking status, and physical activity;

Model 3: Adjusted for covariates in Model 2+ hypertension, T2DM, stroke, heart disease, chronic kidney disease, and cancer.

**P*<0.05

Supplementary Table 4 Association between MetS and/or CircS status and cognitive function (overall and domains) in Chinese middle-aged and elderly participants based on the generalized estimating equation after excluding those diagnosed with brain damage, mental retardation, or memory-related diseases in 2013 or 2015 (N=9,596).

**Cognitive function**	**MetS and/or CircS status**			
	**Normal (n = 5,058)**	**MetS/CircS (n = 4,634)**		
		MetS alone (n = 1,064)	CircS alone (n = 418)	Both MetS and CircS (n = 3,056)
**Executive function**				
Model 1	Ref	0.28 (0.15, 0.41)	**-0.40 (-0.60, -0.19) ***	-0.04 (-0.12, 0.05)
Model 2	Ref	0.08 (-0.12, 0.29)	-0.34 (-0.69, 0.01)	**-0.18 (-0.34, -0.02) ***
Model 3	Ref	0.12 (-0.09, 0.33)	-0.31 (-0.66, 0.04)	-0.14 (-0.30, 0.03)
**Episodic memory**				
Model 1	Ref	0.28 (0.10, 0.45)	**-0.40 (-0.65, -0.14)**	-0.11 (-0.23, 0.01)
Model 2	Ref	0.03 (-0.24, 0.29)	**-0.50 (-0.95, -0.05) ***	**-0.25 (-0.46, -0.04) ***
Model 3	Ref	0.05 (-0.22, 0.33)	**-0.51 (-0.96, -0.06)**	**-0.23 (-0.46, -0.01) ***
**Overall cognitive function**				
Model 1	Ref	0.51 (0.27, 0.76)	**-0.80 (-1.17, -0.44)**	-0.13 (-0.30, 0.04)
Model 2	Ref	0.09 (-0.30, 0.48)	**-0.79 (-1.45, -0.13) ***	**-0.38 (-0.68, -0.08) ***
Model 3	Ref	0.14 (-0.26, 0.54)	**-0.78 (-1.45, -0.11) ***	**-0.34 (-0.66, -0.02) ***

Model 1: Adjusted for follow-up time, age, age^2, gender, residence, educational level, household annual income, and marital status;

Model 2: Adjusted for covariates in Model 1 + body mass index, smoking status, drinking status, and physical activity;

Model 3: Adjusted for covariates in Model 2+ hypertension, T2DM, stroke, heart disease, chronic kidney disease, and cancer.

*P<0.05

Supplementary Table 5 Association between MetS and/or CircS status and cognitive function (overall and domains) in Chinese middle-aged and elderly participants based on the generalized estimating equation after multiple imputation of missing data (N=9,770).

Cognitive function	MetS and/or CircS status			
	Normal (n = 5,058)	MetS/CircS (n = 4,634)		
		MetS alone (n = 1,064)	CircS alone (n = 418)	Both MetS and CircS (n = 3,056)
**Executive function**				
Model 1	Ref	**0.27 (0.14, 0.40) ***	**-0.43 (-0.64, -0.22) ***	-0.04 (-0.13, 0.05)
Model 2	Ref	**0.15 (0.02, 0.28) ***	**-0.46 (-0.66, -0.26) ***	**-0.18 (-0.27, -0.09) ***
Model 3	Ref	**0.17 (0.04, 0.30) ***	**-0.43 (-0.63, -0.23) ***	**-0.14 (-0.24, -0.04) ***
**Episodic memory**				
Model 1	Ref	**0.26 (0.08, 0.44) ***	**-0.45 (-0.70, -0.20) ***	-0.11 (-0.23, 0.01)
Model 2	Ref	0.14 (-0.04, 0.32)	**-0.47 (-0.72, -0.22) ***	**-0.25 (-0.38, -0.12) ***
Model 3	Ref	0.16 (-0.02, 0.34)	**-0.46 (-0.72, -0.20) ***	**-0.22 (-0.36, -0.08) ***
**Overall cognitive function**				
Model 1	Ref	**0.49 (0.24, 0.73) ***	**-0.91 (-1.27, -0.55) ***	-0.13 (-0.30, 0.04)
Model 2	Ref	**0.27 (0.02, 0.52) ***	**-0.95 (-1.32, -0.58) ***	**-0.40 (-0.58, -0.22) ***
Model 3	Ref	**0.30 (0.04, 0.56) ***	**-0.91 (-1.28, -0.54) ***	**-0.34 (-0.53, -0.15) ***

Model 1: Adjusted for follow-up time, age, age^2, gender, residence, educational level, household annual income, and marital status;

Model 2: Adjusted for covariates in Model 1 + body mass index, smoking status, drinking status, and physical activity;

Model 3: Adjusted for covariates in Model 2+ hypertension, T2DM, stroke, heart disease, chronic kidney disease, and cancer.

**P*<0.05

Supplementary Table 6 Association between MetS and/or CircS status and cognitive function (overall and domains) in Chinese middle-aged and elderly participants based on the generalized estimating equation after excluding the participants who had smoking history at baseline (N=5,934).

Cognitive function	MetS and/or CircS status			
	Normal (n = 2,839)	MetS/CircS (n = 3,095)		
		MetS alone (n = 640)	CircS alone (n = 423)	MetS alone (n = 640)
Executive function				
Model 1	Ref	**0.31 (0.14, 0.48) ***	**-0.39 (-0.66, -0.12) ***	0.02 (-0.10, 0.13)
Model 2	Ref	0.11 (-0.08, 0.30)	**-0.46 (-0.75, -0.17) ***	**-0.15 (-0.28, -0.01) ***
Model 3	Ref	0.14 (-0.06, 0.34)	**-0.43 (-0.73, -0.14) ***	-0.13 (-0.27, 0.01)
Episodic memory				
Model 1	Ref	**0.30 (0.07, 0.53) ***	**-0.45 (-0.77, -0.12) ***	-0.14 (-0.29, 0.01)
Model 2	Ref	0.19 (-0.07, 0.45)	**-0.54 (-0.88, -0.19) ***	**-0.37 (-0.54, -0.19) ***
Model 3	Ref	0.21 (-0.05, 0.48)	**-0.52 (-0.88, -0.17) ***	**-0.38 (-0.56, -0.19) ***
Overall cognitive function				
Model 1	Ref	**0.58 (0.24, 0.91) ***	**-0.88 (-1.36, -0.39) ***	-0.14 (-0.36, 0.08)
Model 2	Ref	0.31 (-0.06, 0.68)	**-1.01 (-1.53, -0.50) ***	**-0.50 (-0.75, -0.25) ***
Model 3	Ref	0.36 (-0.02, 0.75)	**-0.96 (-1.49, -0.43) ***	**-0.50 (-0.77, -0.23) ***

Model 1: Adjusted for follow-up time, age, age^2, gender, residence, educational level, household income per year, and marital status;

Model 2: Adjusted for covariates in Model 1 + body mass index, drinking status, and physical activity;

Model 3: Adjusted for covariates in Model 2+ hypertension, T2DM, stroke, heart disease, chronic kidney disease, and cancer.

*P<0.05
